# Reply to “volatile anaesthetics for ICU sedation: beyond hypnosis?”: A comment on “volatile anesthetics for lung- and diaphragm-protective sedation”

**DOI:** 10.1186/s13054-024-05182-w

**Published:** 2024-11-28

**Authors:** Lukas M. Müller-Wirtz, Marcus J. Schultz, Andreas Meiser

**Affiliations:** 1 Department of Anesthesiology, Friedrich-Alexander-Universität Erlangen-Nürnberg, University Hospital Erlangen, 91054 Erlangen, Germany; 2 OUTCOMES RESEARCH CONSORTIUM, Houston, TX USA; 3 Department of Intensive Care, Amsterdam University Medical Center, Amsterdam, The Netherlands; 4 Division of Cardiac Thoracic Vascular Anesthesia and Intensive Care Medicine, Department of Anesthesiology, Intensive Care Medicine and Pain Medicine, Medical University of Vienna, Vienna, Austria; 5 Department of Anesthesiology, Intensive Care and Pain Therapy, Saarland University Medical Center and Saarland University Faculty of Medicine, Homburg, Saarland Germany

We appreciate the comment by Añón and colleagues on our review article, which contributes to the discussion of potential benefits of inhaled sedation beyond hypnosis [1]. One key purpose of review articles is to explore developing areas of research. As Añón and colleagues illustrate well in their comment, our article synthesizes established pharmacological properties of volatile anesthetics with early findings on their use in transitioning critically ill patients to spontaneous ventilation under inhaled sedation, aiming to evaluate potential lung and diaphragm protection benefits [2]. Our focus was on highlighting the importance of gathering detailed clinical data to explore feasibility of lung- and diaphragm-protective ventilation across various sedatives, including volatile anesthetics, rather than drawing any definitive conclusions.

We agree that an increase in instrumental dead space, especially in patients ventilated with lower tidal volumes, where it can add up to 15–30% to the dead space fraction, should be minimized. It is up to the industry to ensure that this consideration is incorporated into further developments.

Completely abolishing respiratory drive with sedation is easy—much easier than titrating sedation and ventilatory support for safe and sufficient spontaneous ventilation. On the one hand, volatile anesthetics provide a broad dose range in which respiratory drive is maintained and can thus be modulated. On the other hand, if return of spontaneous breathing efforts is not a treatment goal, spontaneous breathing activity can be suppressed under inhaled sedation with volatile anesthetics. This becomes apparent from comparing spontaneous ventilation time rates of the multi-center population of the Sedaconda trial with our single-center subgroup [3, 4]. With strongly enforced treatment standards that promote transition to spontaneous ventilation, patients sedated with isoflurane at our center were breathing spontaneously during 82% of time compared to only 42% of time at other centers within the first 20 h after randomization [3, 4].

To promote clarity, we advocate a tailored approach rather than a “one-sedative-fits-all” strategy. Beyond traditional sedation scales, it remains essential to consider how specific sedatives impact respiratory drive and effort to achieve individualized, patient-centered sedation. While inhaled sedation may not suit every patient or situation, it is a valuable addition to our sedation toolbox. Particularly, in patients who require moderate-to-deep sedation but at the same time should transition to spontaneous ventilation, inhaled sedation can be a game changer.

## Data Availability

No datasets were generated or analysed during the current study.
